# Model for analysis of water-cleaning impact on polysilane-based antisoiling coating in PV system

**DOI:** 10.1038/s41598-026-45182-0

**Published:** 2026-04-20

**Authors:** Saheli Sengupta, Deepanjana Adak, Pradipta Sankar Maiti, Aritra Ghosh, Raghunath Bhattacharyya

**Affiliations:** 1 Institute of Engineering and Management, University of Engineering and Management, Kolkata, India; 2 School of Advanced Materials, Green Energy and Sensor Systems, Indian Institute of Engineering Science and Technology, Shibpur, India; 3 Department of Chemistry, Ben-Gurion University of the Negev, 84105 Beer-Sheva, Israel; 4 Faculty of Environment, Science, and Economy (ESE), Renewable Energy,Electric and Electronic Engineering, University of Exeter, Penryn, TR10 9FE UK

**Keywords:** Energy science and technology, Engineering, Environmental sciences, Materials science

## Abstract

Soiling remains a critical global challenge for photovoltaic (PV) systems, reducing power generation and undermining long-term performance. Self-cleaning surface coatings offer a promising mitigation strategy, yet their effectiveness strongly depends on environmental conditions, particularly the availability of water for dust removal. In this paper, development and evaluation of three polysiloxane-based hydrophobic coatings designed to enhance the self-cleaning performance of PV modules is reported. The coatings are synthesized and characterized through optical, morphological, and accelerated UV stability analyses, and their soiling behavior is assessed using an analytical model and year-long field experiments. Model simulations and experimental validation reveal that in the absence of rainfall, fine dust particles (< 5 μm) accumulate similarly on coated and uncoated glass, while larger particles (≥ 5 μm) adhere more readily to coated surfaces. Rainfall, however, substantially improves cleaning efficiency on coated glass, increasing its optical transmittance from 1.1% to 2.24% relative to bare glass. Energy yield modeling for a 10 kWp PV system indicates that the use of the developed coatings can enhance annual power output by up to 6.2%. Seasonal analysis further shows that energy gains following rainfall events can reach 5.1% in wet periods, decreasing to 3.35% during prolonged dry conditions. Techno-economic analysis also indicates the return on investment (ROI) time is much less than any other water-assisted cleaning method. These results demonstrate that hydrophobic polysiloxane coatings can mitigate soiling-induced energy losses and enhance the sustainability of solar power generation, particularly in regions characterized by intermittent precipitation and high dust loads.

## Introduction

Global electricity demand is projected to grow by nearly 4% annually through 2027, equivalent to an additional ~ 3500 TWh each year, largely driven by industrialization and electrification of end-use sectors^1^. Solar photovoltaics (PV), surpasses 2.2 TWh at end of 2024 indicating its sharp growth among all other renewable sources^2^. Currently solar PV technologies include first generation silicon, second generation thin film and third generation dye-sensitized solar cell **(**DSSC), Perovskite and organic types^3–5^. Due to the efficiency limit of single junction solar cells, tandem structures are also in the consideration^6^. However, silicon technology continues to dominate the photovoltaic market due to its long-term stability and moderate-to-high efficiency. Nevertheless, when these solar cells are deployed outdoors as modules, their performance is influenced by varying weather conditions and operational parameters which can reduce their ability to harness maximum solar radiation, thereby affecting overall energy yield. One of the most critical factor is soiling which becomes a global issue creating energy and economic loss in PV sector. In the long-term, soiling may cause hot-spot generation on modules due to non-uniform heating which ultimately leads to reduce their life^7^.

It has emerged as the second most detrimental factor after irradiance, leading to global energy losses of 3–5% annually, with associated economic consequences estimated at €3–5 billion per year^8,9^. Extensive investigations have characterized soiling in desert and semi-arid regions, where deposition patterns are comparatively predictable^10,11^. However, the Indian subcontinent remains understudied despite its rapidly expanding solar installations. This region is characterized by high humidity, seasonal monsoons, and diverse pollution sources that influence both deposition dynamics and particle adhesion mechanisms differently than in arid climates^8,12^.

Recent experimental studies in Bangladesh demonstrated that PV efficiency losses from soiling persist even under frequent rainfall, with degradation strongly linked to pollution levels and cleaning intervals^13^. A broader South Asian analysis further revealed that soiling losses in humid and semi-humid climates range from minor to severe, emphasizing the necessity for location-specific mitigation strategies^14,15^. Despite these findings, a critical knowledge gap persists. Anti-soiling coatings and treatments effective in desert conditions have not been validated in the Indian subcontinent. Antisoiling coatings show promise under arid environments but may underperform under humidity-driven cementation and biological fouling typical of monsoon climates^9,12^. Addressing this performance degradation is essential for safeguarding PV productivity and reducing the Levelized Cost of Electricity.

Solar energy devices like PV panels and concentrated solar power (CSP) systems require regular cleaning for optimal performance, which can be costly and challenging in remote or inaccessible locations. Various dust-removal methods have been explored in the literature, including wind-assisted self-cleaning, water assisted self-cleaning-antisoiling coating, electrodynamic screens, manual or robotic water cleaning, and air-blast cleaning for water-scarce regions^16^. However, most of these techniques are either labor-intensive, water-intensive, or economically unviable for large-scale deployment. By contrast, anti-soiling coated glass offers the potential for reduced cleaning frequency, extended module life, and improved cost-effectiveness^17^.

Achieving effective self-cleaning coatings requires surfaces with low surface free energy and high optical transparency^16^. Currently they are hydrophilic and hydrophobic types. Hydrophobic coatings inspired by the ‘Lotus Effect’ enable rolling water droplets to carry away particulates. Various artificial self-cleaning surfaces have been developed inspired by this effect^18^. However, balancing anti-reflective and self-cleaning properties remains challenging^19^. Maintaining the dual-scale hierarchical roughness necessary for water repellence against compressive and abrasive forces is particularly difficult, as induced porosity in the coating improves transparency but reduces hardness^20^. Long-term operation of solar systems also requires stable, self-cleaning technologies that maintain optical properties despite environmental conditions. Most outdoor experimental work on anti-soiling coatings has been conducted in low-humidity, high-dust regions such as the Middle East and North Africa^21,22^.

Quan et al.^23^ experimentally tested transparent hydrophobic coatings on solar cell cover glass by measuring contact angles, optical transmittance, and dust deposition. The coated glass showed high hydrophobicity (contact angle > 100°), minimal light loss (< 2%), and about 40–60% less dust accumulation than uncoated glass. Water-drop tests confirmed good self-cleaning performance, demonstrating that such coatings effectively reduce soiling while maintaining optical efficiency. Bekir Sami Yilbas et al.^24^ uses high-speed imaging and detailed surface characterization to analyze water droplet impact, spreading, recoiling, and detachment behaviours for dust removal under different hydrophobic surface conditions. Through a combination of experimental analysis and numerical modelling^25^, the study examines droplet impact, spreading, and rebound dynamics under varying surface wettability and dust conditions. The results show that superhydrophobic surfaces enable droplets to roll and coalesce effectively, reducing dust adhesion and promoting efficient self-cleaning. Lu and Liang^26^ experimentally analyzed how self-cleaning hydrophobic coatings influence dust deposition on solar PV glass under varying conditions. A detailed review on different types of coating to enhance PV performance has been presented in^27^. The study reveals that while hydrophobic coatings markedly reduce soiling and transmittance loss, their performance diminishes with adhesive or moist dust, highlighting the need for environment-specific coating optimization. A summary of available predominant literature on the efficacy study of antisoling coatings is presented in Table 1.

**Table 1:** Summary of literature of performance study of anti-soiling coating.

Study / Author (Year)	Type	Coating type	Test condition	Key performance findings	Notes / Remarks
Walz et al. (2023)^28^	Field	Si/Ti Mixed Oxide Thin Film	Outdoor (U.S. Midwest, 6 months)	Soiling rate reduced by 30–40%; power loss cut by 4%	Strong field validation of hybrid coating
Denmark Field Exposure Study (2020–2024)^29^	Field (long-term)	Fluoropolymer / Silica-based hydrophobic	Outdoor 5-year durability test	Initial 3–6% transmittance gain, but 50% performance loss after 3 years	Shows long-term degradation issues
*Enhanced Dust Reduction Method for Solar Panels* (*Sci. Reports*, 2024)^30^	Lab + Field	Dual-Layer Hybrid	Desert & coastal test sites	39% improved dust repellence vs. uncoated	Promising hybrid performance
IEA-PVPS Multi-Site Coupon Study (2024)^31^	Field	Various commercial coatings	Desert / Humid / Coastal	0–6% power gain; site-dependent	International benchmark study
Hameed et al., *PLOS ONE* (2024)^32^	Comparative	Nano-Coating vs. Mechanical cleaning	Lab + Field	Coatings achieved 85–90% cleaning efficiency vs. 100% for wiper system	Highlights tradeoff between passive and active systems
Lu et al. (2023)^33^	Lab	Super-Hydrophobic (Nano-SiO₂ / Fluorine)	Wind + Wet test rig	Best at tilt angles > 30°; ~25% dust reduction	Emphasizes role of tilt and wind speed

From the reviewed literature, it is evident that researchers have developed various types of anti-soiling coatings to enhance the self-cleaning performance of PV modules operating in dusty environments. Under field conditions, these coatings generally demonstrate improved performance; however, they also encounter significant challenges due to climatic stressors such as temperature fluctuations, humidity, and UV exposure. Several studies have attempted to examine the influence of environmental parameters on the cleaning efficiency of these coatings using artificial dust deposition experiments. Nevertheless, only a limited number of investigations have simultaneously simulated the combined effects of rainfall or water impact and other environmental stressors on the effective self-cleaning behavior of PV modules at a particular location.

In this study, a physics-based model is employed to analyze the water-based dust removal performance of a laboratory-developed polysiloxane-based hydrophobic anti-soiling coating. The model predictions are validated through one-year outdoor exposure tests conducted on coated PV glass coupons. A comprehensive comparative analysis between coated and bare glass is performed for all investigated cases. The contributions of the work are as follows:

- Three types of polysiloxane-silica nanocomposite are designed and developed for hydrophobic, oleophobic, and anti-reflective performance on solar glass.
- Different characterization and UV accelerated testing are carried out to choose and justify the best one.
- Field experiment with and without coatings on PV glass is carried out over a period of one year to substantiate the efficacy of the coatings with periodic monitoring of transmittance variation.
- An analytical approach is utilised to simulate the performance of the developed coatings as compared to uncoated sample for dust deposition under various climatic conditions. It has also been extended to study the impact of water-based cleaning for both types of PV glasses.
- Finally, assessment of soiling loss reduction with the developed antisoling coating is also carried out based on the optical approach and compared with that of uncoated one for one year.
- An economic feasibility study for an existing power plant has been examined.

The assumptions made in this study are:

(i) The particles are spherical in shape.
(ii) Only concentration of PM_2.5_ and PM_10_ are considered to calculate dust accumulation on modules.
(iii) Wavelength dependant spectral transmission is not considered here in PV generation assessment.
(iv) Silica is only considered here as it is maximum in percentage among all other constituent^34^ at the place of experimentation.
(v) Cementation and biological fouling are not considered in this analysis.

Section Methodology describes different steps of the coating development, their performance study, field experimentation and model development. Field results including cost benefit analysis are carried out in Section  Result and discussion followed by a concluding remark in Section Economic analysis.

## Methodology

**Fig. 1:**
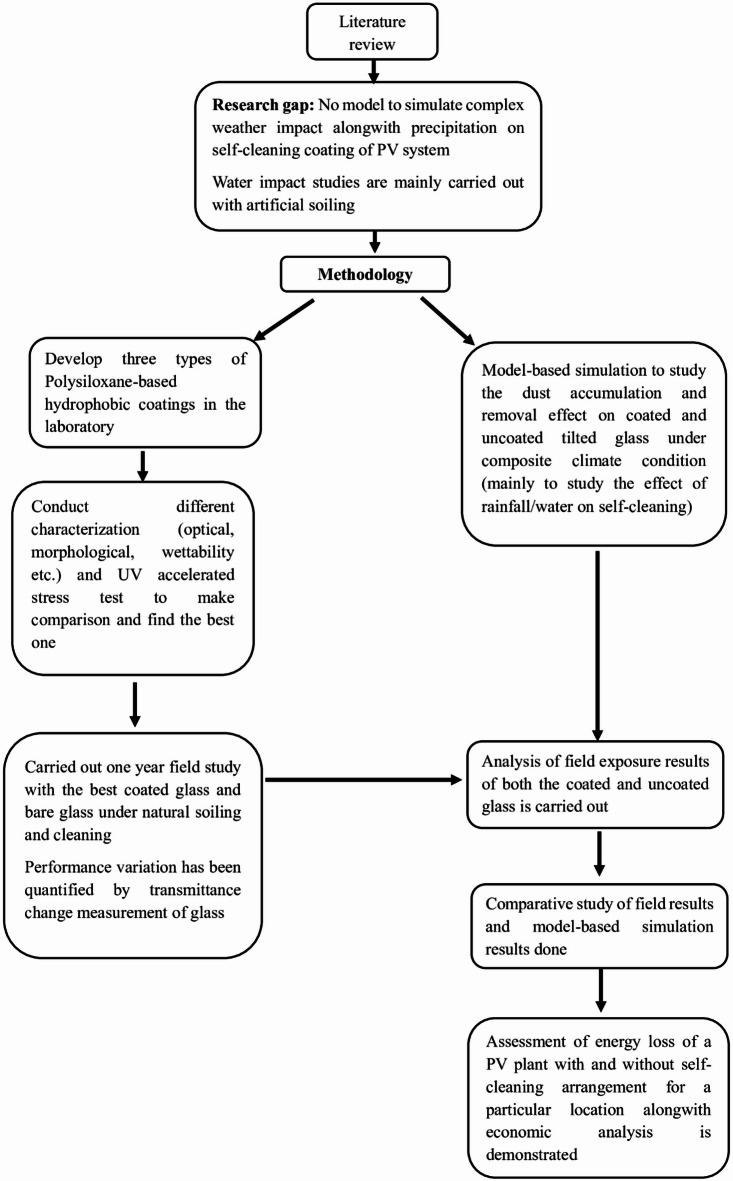
Flow diagram of the investigated work.

The overall workflow is illustrated in Fig. 1.

### Experimental details of coating development

The coating evaluated in this study was developed as a polysiloxane-silica nanocomposite designed for hydrophobic, oleophobic, and anti-reflective performance on low iron solar glass. The formulation and detailed synthesis methodology are described in the Indian Patent by the authors^35^. The coating composition comprised organically modified siloxane precursors combined with colloidal silica nanoparticles (C20DH and C30DH), both procured commercially in ethanol dispersions. Analytical-grade isopropanol, ethanol, acetone, and hydrochloric acid were obtained from Merck & Co. and used without further purification. Hexamethyldisilazane (HMDS) was employed as a post-treatment agent to enhance surface hydrophobicity, while deionized (DI) water was utilized throughout the cleaning and solution preparation processes. Prior to coating, the glass substrates were subjected to a multi-step cleaning protocol comprising mild mechanical brushing, alkaline detergent sonication, degreasing in acetone and isopropanol, and thermal pre-treatment at 100°C. The coating solution was prepared by dissolving trimethoxymethylsilane (TMS) in isopropanol, followed by the dropwise addition of silica nanoparticle dispersions under continuous stirring to ensure homogeneous mixing. The resulting solution was aged for 24 h to complete hydrolysis and condensation reactions. Coatings were deposited using a dip-coating process with controlled withdrawal speeds (50–100 mm/min), enabling systematic optimization of film thickness and surface properties. Post-deposition, the coated glass was thermally cured at 100–120 °C to ensure crosslinking and strong adhesion. A final surface treatment with 2% HMDS solution was applied to further reduce surface energy and improve dust-shedding performance.

### Performance analysis of developed coating

The polysiloxane-silica nanocomposite coating was extensively characterized to evaluate its suitability for PV applications in humid environments. The results demonstrate that the integration of silica nanoparticles into the polysiloxane matrix significantly enhances optical, surface, and durability properties compared to unmodified coatings, particularly enhancing the efficacy of water-based cleaning leading to soiling loss mitigation.

#### Optical performance

**Fig. 2:**
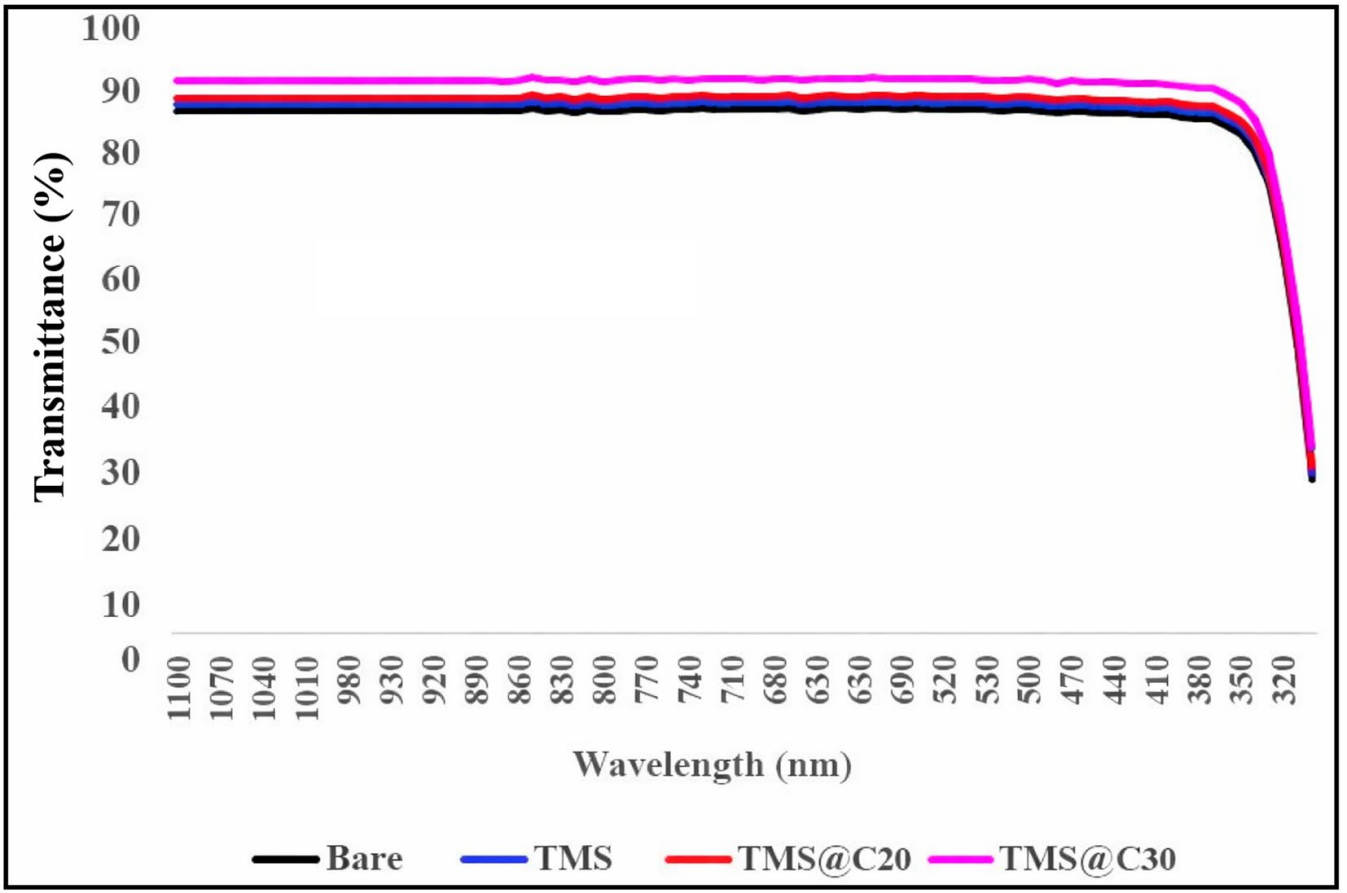
Plot of optical transmission (%) vs. wavelength (nm) of polysiloxane coatings.

**Fig. 3:**
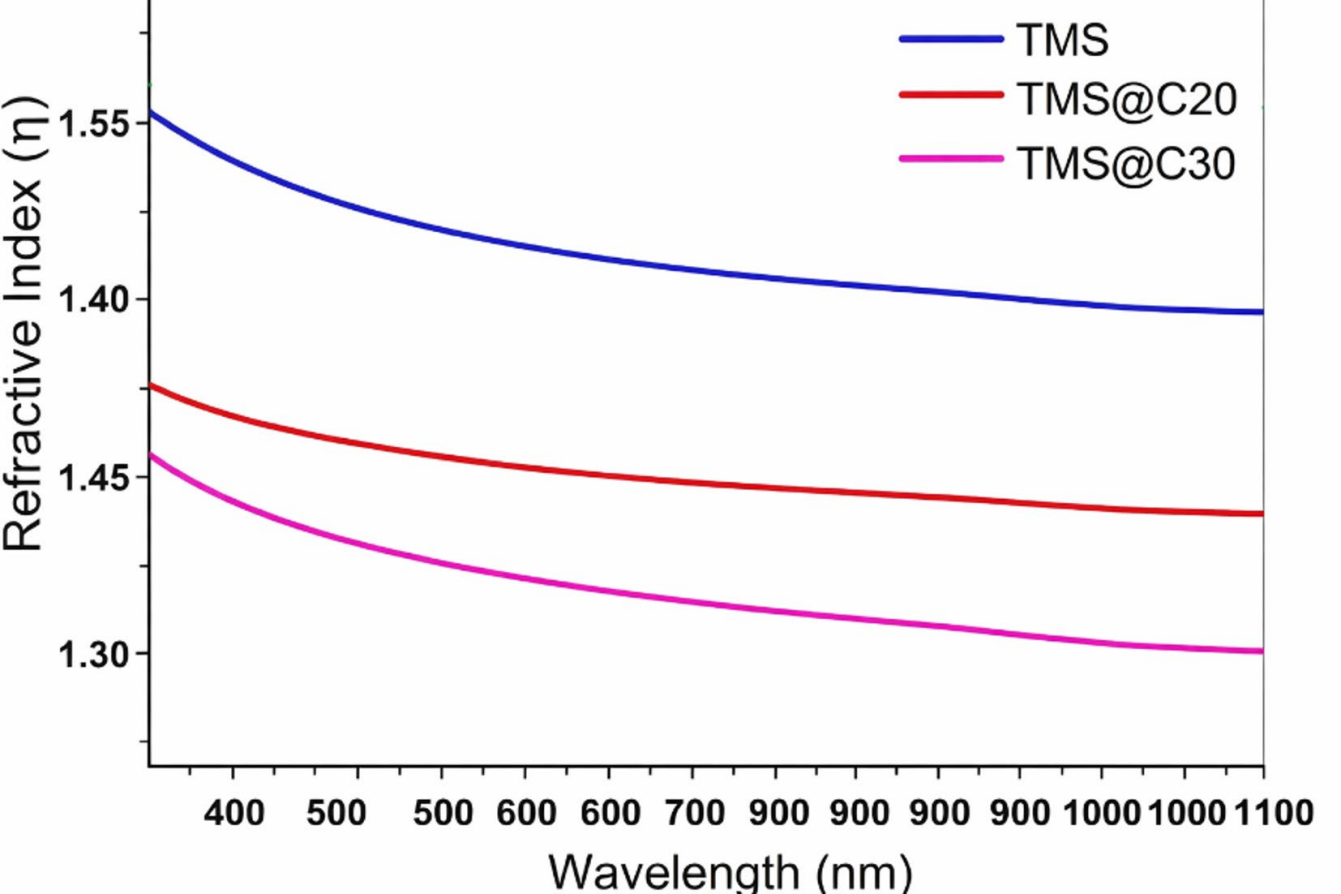
Plot of refractive index (optical constant) vs. wavelength (nm) of all the polysiloxane.

The transmission spectra (Fig. 2) reveal that all coated glass samples exhibit high transmittance in the visible to near-infrared range (400–1100 nm), for nanoparticle-modified coatings. The incorporation of C20 and C30 silica nanoparticles reduced scattering losses and minimized reflection, resulting in a more uniform spectral response across the solar-relevant wavelength range. The enhanced optical performance is consistent with the lower refractive indices measured via spectroscopic ellipsometry as shown in Fig. 3, where the TMS@C30 coating demonstrated the most favourable value of refractive index (RI) 1.37 at 637 nm (as shown in Table 2), confirming effective refractive index tuning through nanostructuring.

**Table 2:** Optical constants and physical parameters of different polysiloxane coatings.

Sample label	Thickness (nm)	Refractive index (637 nm)
TMS	90	1.51
TMS@C20	89	1.47
TMS@C30	58	1.37

#### Chemical structure and surface functionality

**Fig. 4:**
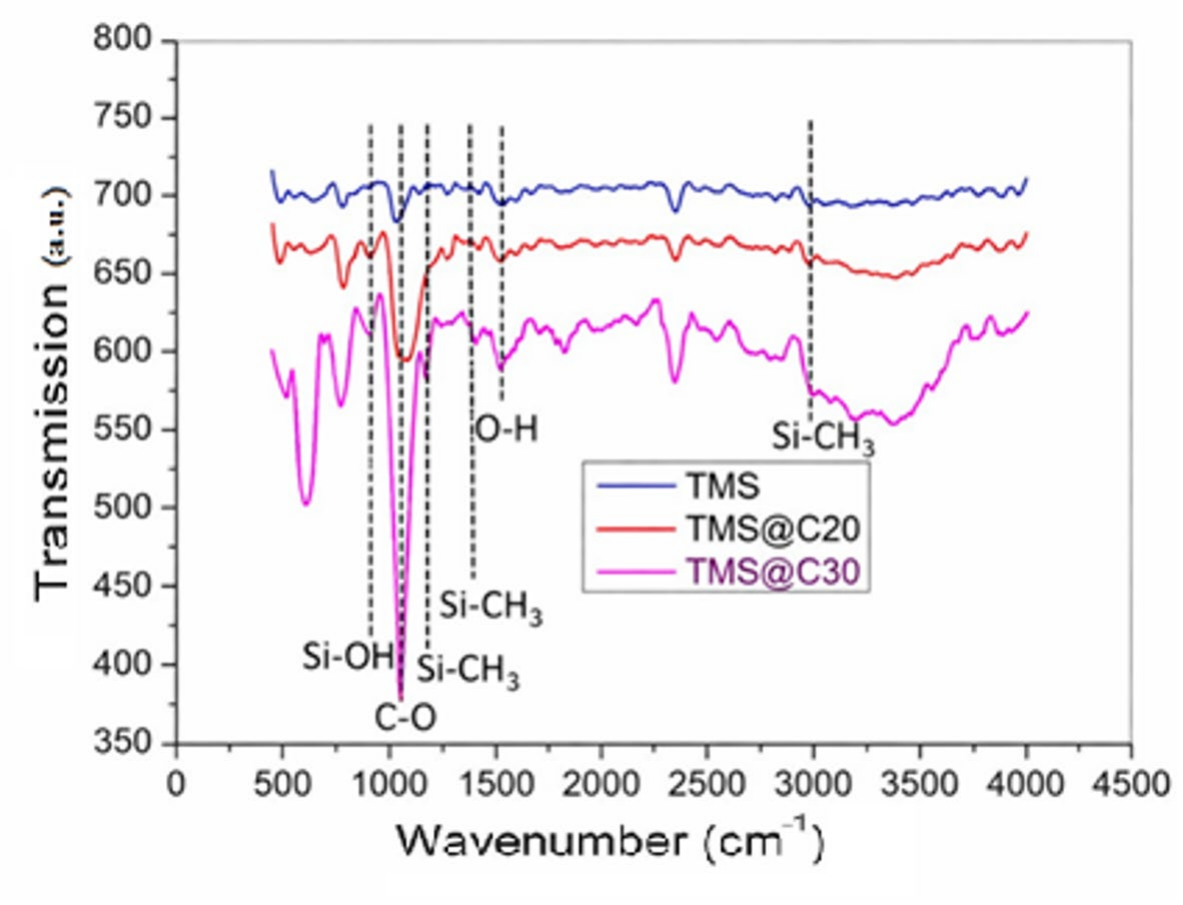
FTIR spectrum in transmission mode of various polysiloxane coating with and without silica nanoparticles.

Fourier-transform infrared (FTIR) spectroscopy (Fig. 4) confirmed the successful formation of a crosslinked polysiloxane network. Characteristic Si–O–Si stretching vibrations were observed, alongside features attributed to methyl functionalities, which are essential for imparting hydrophobicity. The presence of these functional groups, combined with the incorporation of silica nanoparticles, results in a stable, low-energy surface optimized for anti-soiling performance.

#### Surface morphology and wettability

**Fig. 5:**
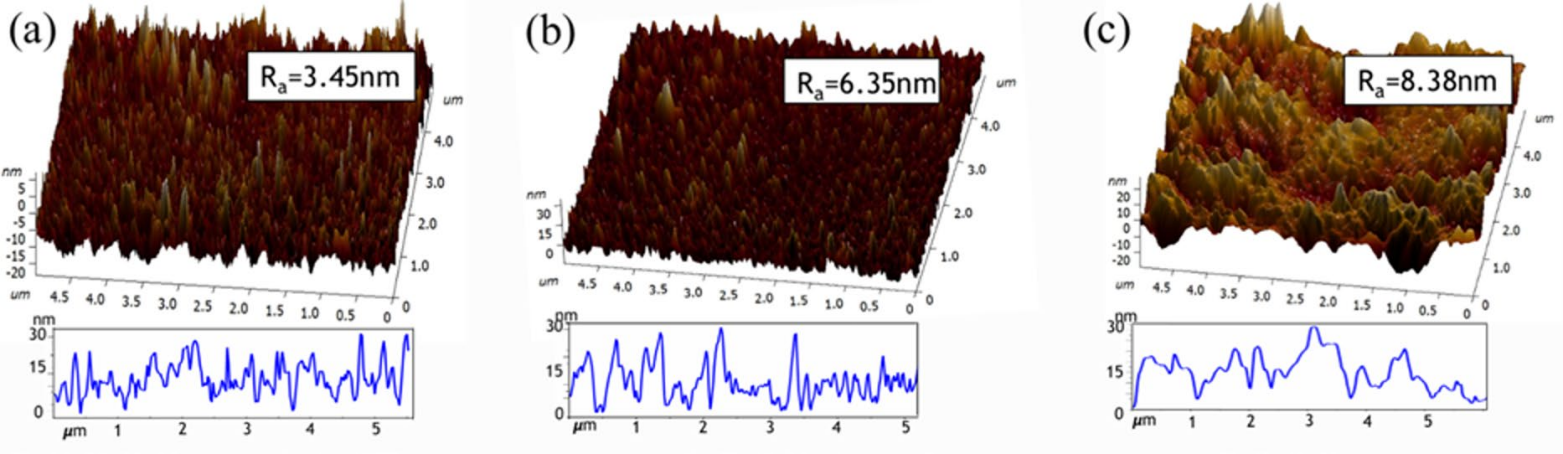
3D AFM and corresponding 1D profile topography images (approximate image size ~ 5 × 5 μm) of different polysiloxane coating with RMS roughness of each type of polysiloxane coating shown in the inset (**a**) TMS, (**b**) TMS@C20, and (**c**) TMS@C30.

**Fig. 6:**
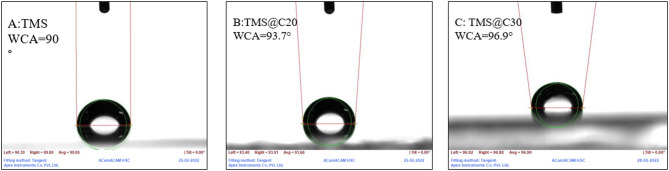
Water contact angle measurement of three different coated samples.

Atomic force microscopy (AFM) revealed that coatings incorporating nanoparticles possess a finely textured, uniform surface with root mean square (RMS) roughness between 6 and 9 nm, considerably higher than unmodified polysiloxane films as shown in Fig. 5. This dual-scale nanoscale roughness plays a pivotal role in promoting water repellence, as evidenced by contact angle measurements (Fig. 6). The TMS@C30-coated samples exhibited water contact angles of ~ 97° with low hysteresis (Table 3), ensuring efficient roll-off of water droplets and removal of adhered particulates during rainfall events. Correspondingly, low surface free energy values were obtained, further substantiating the coating’s self-cleaning capability.

**Table 3:** Contact angle and surface free energy measurement results of different polysiloxane coatings.

Sample label	Water contact angle (degree)	Diiodomethane contact angle (degree)	Contact angle hysteresis	Surface free energy (mN/m)
TMS without nanoparticle	90	51	21	34.6
TMS@C20	93.7	69	9	28
TMS@C30	97	75	10	25

#### Mechanical integrity and environmental stability

**Fig. 7:**
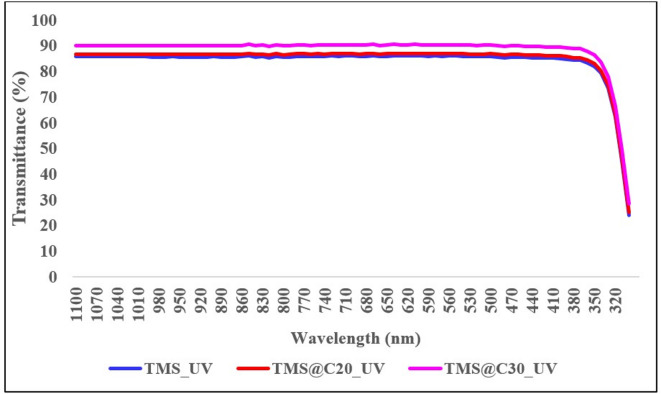
Variation of transmittance of coated glass samples with wavelength under UV exposure.

Mechanical robustness, essential for outdoor PV applications, was confirmed through standardized testing and the test results are given in Table 4. The coatings exhibited pencil hardness up to 6 H (ASTM D3363) and excellent adhesion (ASTM D3359, 5B rating), indicating strong resistance to abrasion and delamination. Accelerated ultraviolet (UV) exposure tests under combined UVA and UVB irradiation (15 kWh/m² at 60 ± 5 °C) showed optical degradation for three samples, with integrated transmittance losses limited to 1.2% (TMS@C30), 1.9% (TMS@C20) and 2.5% (TMS), demonstrating long-term stability against photodegradation and environmental stress. Figure 7 shows the spectral response of the transmittance change of coated and bare glasses before and after UV aging test.

**Table 4:** Pencil hardness and crosshatch cut adhesion test value of the coatings.

Sample level	Pencil hardness	Adhesion
TMS without nanoparticle	7 H	5B
TMS@C20	6 H	5B
TMS@C30	6 H	5B

### Field experimentation

**Fig. 8:**
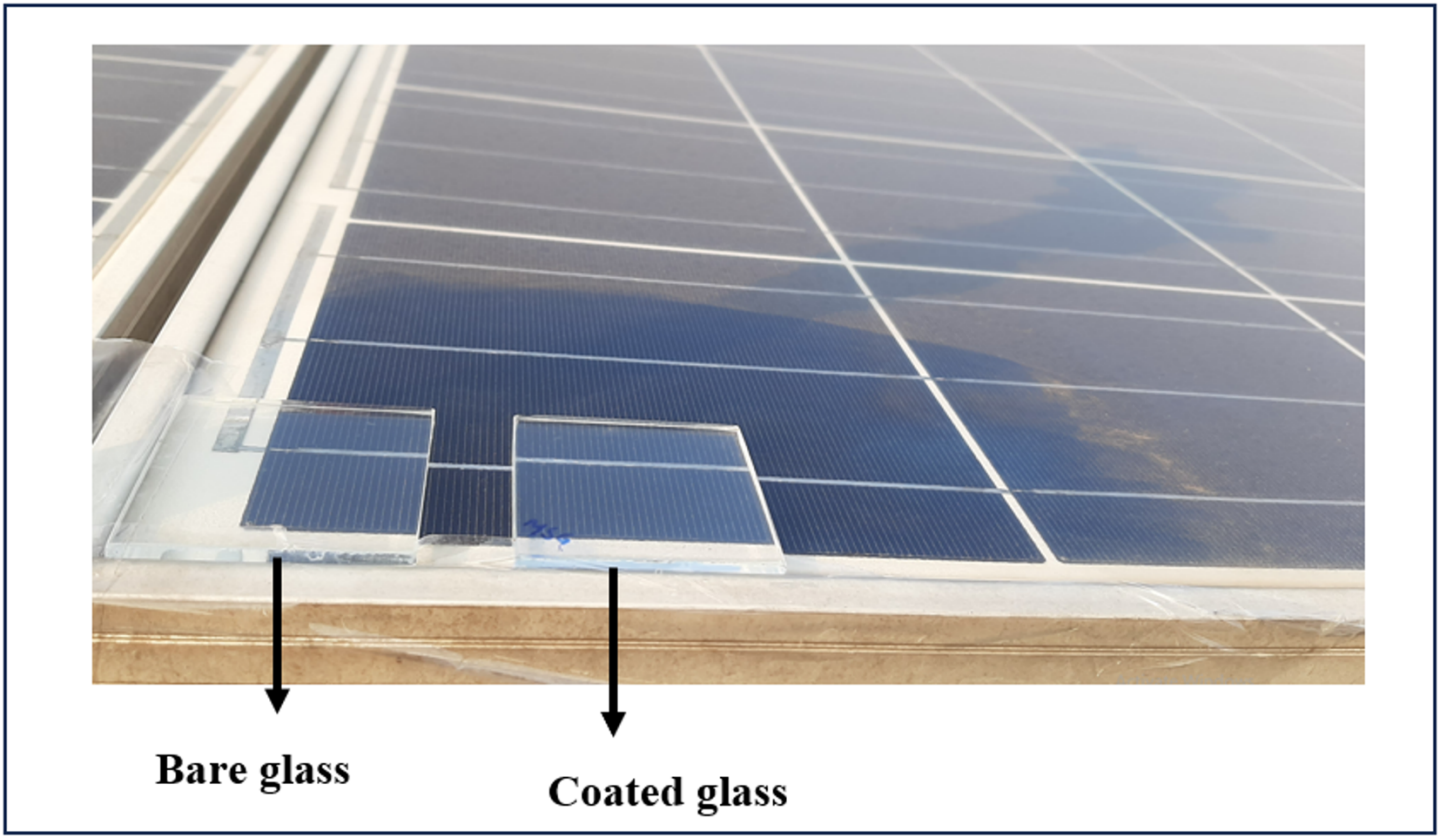
Bare and coated glass coupons are kept in outdoor environment.

From the characterization and accelerated UV test, it is found that the glass coated with TMS@C30 provides the best result. Therefore, low iron glass coupons of size 5⋅5 cm^2^ with this particular coating are placed on the modules of a roof-top PV plant at 25º tilt angle for one-year period (November, 2021 to November, 2022) along with uncoated glass coupons as shown in Fig. 8. For a specific interval (once in two weeks), the glass coupons are taken from the module surface for the measurement of its change in transmittance by UV-Vis spectrophotometer and then kept in the same position. Comparison in weight variation due to dust accumulation for bare as well as coated glass are also measured using Quintix 125D-1S semi-Microbalance. The size distribution of dust particle in various seasons is also recorded by optical microscopic analysis, Optika B-600 MET microscope with an image analyzer. Weather data and PM concentration data are collected from Solar Radiation Resource Assessment (SRRA) station installed at IIEST Shibpur and Central Pollution Control Board (CPCB) website.

### Modeling of dust deposition and removal

To understand the coating’s performance under real-world conditions, a physics-based model is developed^36^ to simulate the competing processes of dust deposition and removal on coated and uncoated glass surfaces. This will help to find the correct coating in PV industry to maximize the energy output in a particular environment. The model considered turbulent and Brownian diffusion, gravitational settling, particle impaction, and adhesion effects influenced by relative humidity, along with natural cleaning forces from wind and rainfall. Simulations predicted that while initial dust deposition rates on coated and uncoated surfaces were comparable, the coated surfaces demonstrated significantly higher removal efficiency during rain events, reducing net soiling accumulation over time. The model outputs correlated strongly with experimental observations, validating its predictive capability.

#### Modeling of dust deposition

A two-layer (e.g. turbulent layer and laminar layer) dust deposition model is considered to assess the amount of particle deposition in outdoor condition. From the turbulent layer particles are dropping downwards due to eddy diffusion and gravitational settling. From the near surface or laminar layer, particles move due to molecular diffusion and impaction processes. Combining all these phenomena, deposited dust mass $\:{M}_{d}$ in presence of RH on glass surface can be estimated by (1)^36^,

1 $$\:{M}_{d}= \left[\frac{B\:\mathrm{e}\mathrm{x}\mathrm{p}(-B\:{A}_{int}/{u}_{*})}{1-\mathrm{e}\mathrm{x}\mathrm{p}(-B\:{A}_{int}/{u}_{*})} \right]\:{{C}_{PM}t}_{d}A$$

First part of (1) determines the velocity of dust particle deposition including particles’ terminal velocity $\:{V}_{t}$, and impaction velocity $\:{V}_{im}$,

2 $$\:B=({V}_{t}cos\theta\:+{V}_{im})$$

3 $$\:{V}_{im}=\frac{{A}_{im}{U}_{w}{\:\:\sigma\:}_{dir}}{1+{e}^{-\left(f\right({S}_{tk}-1\left)\right)}}$$

4 $$\:\mathrm{a}\mathrm{n}\mathrm{d},\:{A}_{int}=\:{\int\:}_{\frac{Z{u}_{*}}{\vartheta\:}}^{\frac{{2r}_{P}{u}_{*}}{\vartheta\:}}\frac{d\left(\frac{Z{u}_{*}}{\vartheta\:}\right)}{\frac{e}{\vartheta\:}+\frac{D}{\vartheta\:}}$$

where, $\:{r}_{P}$ is the particle radius in presence of RH, $\:{u}_{*}$ is the wind friction velocity, $\:\vartheta\:$ is the dynamic viscosity of air, $\:e$ and $\:D$ are the eddy and Brownian diffusivity, $\:\theta\:$ is the tilt angle of module, $\:Z$ is the surface height, $\:{\sigma\:}_{dir}$ is the factor of the wind speed that hits the module front surface perpendicularly, $\:{A}_{im}$ is a impaction weighting factor, $\:f$ is estimated from measured data, and $\:{S}_{tk}$ is the Stokes number, $\:A$ is the area of dust deposition, $\:{t}_{d}$ is time of deposition, $\:{C}_{PM}$ is the PM concentration of particulate matter in atmosphere.

#### Modeling of dust removal

Wind force plays a dual role in the dust deposition process: on one hand, it facilitates the accumulation of dust on surfaces, while on the other, it aids in the removal of dust particles^37^. In this study, wind force is represented as, $\:{F}_{w}$ by (5)

5 $$\:{F}_{w}={\frac{1}{2}C}_{x}{\rho\:}_{s}{S}_{p}{{U}_{w}}^{2}$$

where, $\:{\rho\:}_{s}$ is particle density for silica 2600 kg/m^3^^34^, drag coefficient $\:{C}_{x}$, $\:{S}_{p}$ is particles’ area opened to the air.

In a humid environment, a particle can detach from surface only when it can overcome the particle-surface adhesion force^38^. The adhesion force $\:{F}_{ad}$ between a particle and a surface is the summation of Van der Waals force ($\:{F}_{vw}$), capillary force ($\:{F}_{cap}$), surface tension force $\:{(F}_{su)}$, and electrostatic force (neglected here)^39^. Relative humidity (RH) influences this adhesion by enhancing capillary forces while reducing particle re-entrainment rates^40,41^. Capillary force can be calculated as (6),

6 $$\:F_{{cap}} = \:\:\pi \:\gamma \:_{{su}} r_{p} \:\left( { - sin\alpha \:_{c} + \frac{{{\mathrm{cos}}\left( {\theta \:_{{lp}} + \alpha \:_{c} } \right) + cos\theta \:_{{ls}} }}{{H/r_{p} + 1 - {\mathrm{cos}}\alpha \:_{c} }}{\mathrm{sin}}^{2} \alpha \:_{c} } \right)$$

where, $\:{\gamma\:}_{su}$ is the surface tension of liquid (N/m). $\:{\alpha\:}_{c}$ (rad) is filling angle, $\:H$ is the distance between particle and surface, $\:{\theta\:}_{lp}$ liquid-particle angle and $\:{\theta\:}_{ls}$ is the liquid-surface angle.

Van der Waals force ($\:{F}_{vw}$)^39^ is calculated as (7),

7 $$\:{F}_{vw}=\:\frac{{A}_{w}{r}_{p\:}}{6{H}^{2}}\left\{1-\frac{1}{{{\varDelta\:}_{w}}^{2}}\right\}+\:\frac{{A}_{a}{r}_{p\:}}{6{H}^{2}}\left\{\frac{1}{{{\varDelta\:}_{w}}^{2}}\right\}$$

where, $\:{\varDelta\:}_{w}=$ $\:\left[1+{r}_{p\:}\left(1-cos{\alpha\:}_{c}\right)/H\right]$, $\:{A}_{w}$ and $\:{A}_{a}$ are the Hamaker constant with water and air as medium^39^.

Force due to surface tension ($\:{F}_{su}$) is presented in (8),

8 $$\:{F}_{su}=2\pi\:{\gamma\:}_{su}{r}_{p\:}sin{\alpha\:}_{c}\mathrm{sin}\left({\theta\:}_{lp}+{\alpha\:}_{c}\right)$$

Additionally, the impact of precipitation is considered in the particle detachment process. The force exerted by rainfall, as described in^42^, is calculated using (9),

9 $$F_{r} = \frac{{P_{r} }}{{V_{{t\_r}} }}$$

Terminal velocity of the rainfall $\:{V}_{t\_r}$ is calculated as,

10 $$\:{V}_{t\_r}={\left(\frac{{\left(2{R}_{w}\right)}^{2}{\rho\:}_{W}g}{6\:{K}_{r}}\right)}^{1/3}$$

The coefficient $\:{K}_{r}$ remains constant within a specific range of raindrop diameters, as reported in^43^, raindrop radius is R_w_. The power of the rainfall, denoted as $\:{P}_{r}$ over the duration $\:{t}_{rain}$ is expressed as,

11 $$\:{P}_{r}={E}_{r}{t}_{rain}$$

Energy of precipitation expressed by $\:{E}_{r}$ can be calculated as (12),

12 $$\:{E}_{r}=1288.17\:{{\mu\:}_{w}}^{-1.34}{{I}_{r}}^{1+1.34{\beta\:}_{w}}$$

where, $\:{\mu\:}_{w}$ and $\:{\beta\:}_{w}$ describe the process of growth of raindrops. $\:{\mu\:}_{w}$ is in between 30 and 40 according to process of growth of raindrops, $\:{I}_{r}$ rainfall rate.

Contribution of rainfall force is included to the drag and pull-off forces during wet deposition as mentioned in (13)–(14)

13 $$\:{F}_{drag\_w}=\left(mg\:\mathrm{s}\mathrm{i}\mathrm{n}{\uptheta\:}+{F}_{w}cos\phi\:\right)+{F}_{r}\mathrm{c}\mathrm{o}\mathrm{s}{\uptheta\:}$$

14 $$\:{F}_{at\_w}={\mu\:}_{f}\left((\left({F}_{ad}+mg\mathrm{c}\mathrm{o}\mathrm{s}{\uptheta\:}\right)+{F}_{w}sin\phi\:\right)+{F}_{r}\mathrm{s}\mathrm{i}\mathrm{n}{\uptheta\:})$$

The value of $\:{\mu\:}_{f}$ reaches its maximum under dry conditions and decreases under wet conditions; however, in the ‘tacky condition’, it exceeds the value as observed in dry conditions^44,45^. $\:\phi\:$ is the wind’s angle of attack on a particle.

Therefore, the particle remaining on the surface after resuspension is calculated as,

15 $$\:{f}_{re\_w}=\mathrm{e}\mathrm{x}\mathrm{p}(-{f}_{o\_w}\mathrm{e}\mathrm{x}\mathrm{p} \left [-{\left(\frac{{F}_{at\_w}}{{F}_{drag\_w}}\right)}^{{x}_{f}}{t}_{rain} \right]$$

where, $\:{x}_{f}$ and $\:{f}_{o\_w}$ are constants where $\:{f}_{o\_w}$ depends on frequency of vibration, and value of $\:{x}_{f}$ is taken from^37^.

Therefore, the amount of dust accumulation can be calculated as (16),

16 $$\:{M}_{s}={M}_{d\:}{f}_{re\_w}$$

Dust accumulation on solar PV module glass covers reduces light transmittance, leading to lower energy output^46^. This can be calculated in terms of mass of dust deposited on the module.

17 $$\:{T}_{r}=\mathrm{e}\mathrm{x}\mathrm{p} \left(-\frac{3*\epsilon*{M}_{s}}{4\:{\rho\:}_{s}A{r}_{p}cos\theta\:cos\xi\:}\right)$$

where, $\:\epsilon$ is the transmittance of single layer dust^47^, $\:\xi\:$ is angle of incidence (AOI). Power generation for soiled PV module is determined by modifying Anderson’s model^48^ as (18)–(20).

18 $$\:{v}_{o}={v}_{ref}\left(1+{a}_{v}\left({T}_{mod}-{T}_{ref}\right)\right)(1+{a}_{e}(\mathrm{l}\mathrm{o}\mathrm{g}\left(\frac{E*{T}_{r}}{{E}_{ref}}\right)\left)\right)$$

19 $$\:{i}_{o}={I}_{ref}\left(1+{a}_{i}\left({T}_{mod}-{T}_{ref}\right)\right)\left(\frac{E*{T}_{r}}{{E}_{ref}}\right)$$

20 $$\:{P}_{mod}={n\:v}_{o}{i}_{o}FF$$

where, $\:{P}_{mod}$, is the power output from modules, number of PV module $\:n$, $\:FF$ fill factor, $\:{v}_{ref}$ and $\:{i}_{ref}$ are PV module rated voltage and current respectively, $\:{a}_{v}$ voltage-temperature coefficient, and $\:{a}_{i}$ current-temperature coefficient, and $\:{a}_{e}$ is the radiation-temperature coefficient, $\:E$ amount of total incident radiation (W/m^2^) and $\:{E}_{ref}$ is 1000 W/m^2^ and $\:{T}_{ref}$ is 25 °C, T_mod_ is the module temperature.

## Result and discussion

### Results of model-based analysis

**Fig. 9:**
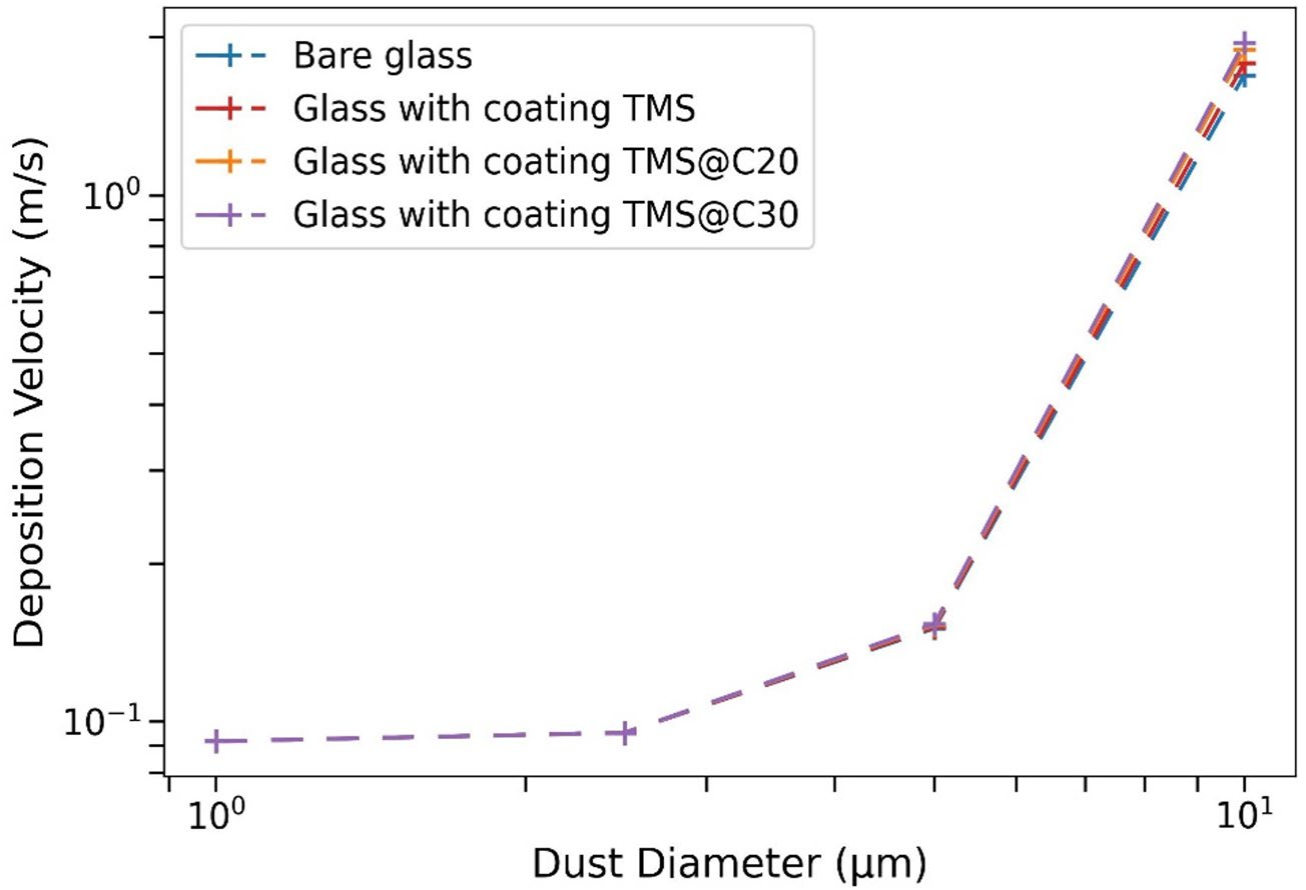
Dust deposition velocity for different diameter particle on three types of coated and uncoated glass.

Using the model, deposition velocity of particles on different samples is calculated for various dust diameters which is presented in Fig. 9.

It is observed in Fig. 9 that, deposition velocity of dust particle at the surface of TMS@C30 coating is higher for large particles and closer for small particles as compared to bare glass surface, as surface roughness is lower for uncoated glass.

**Fig. 10:**
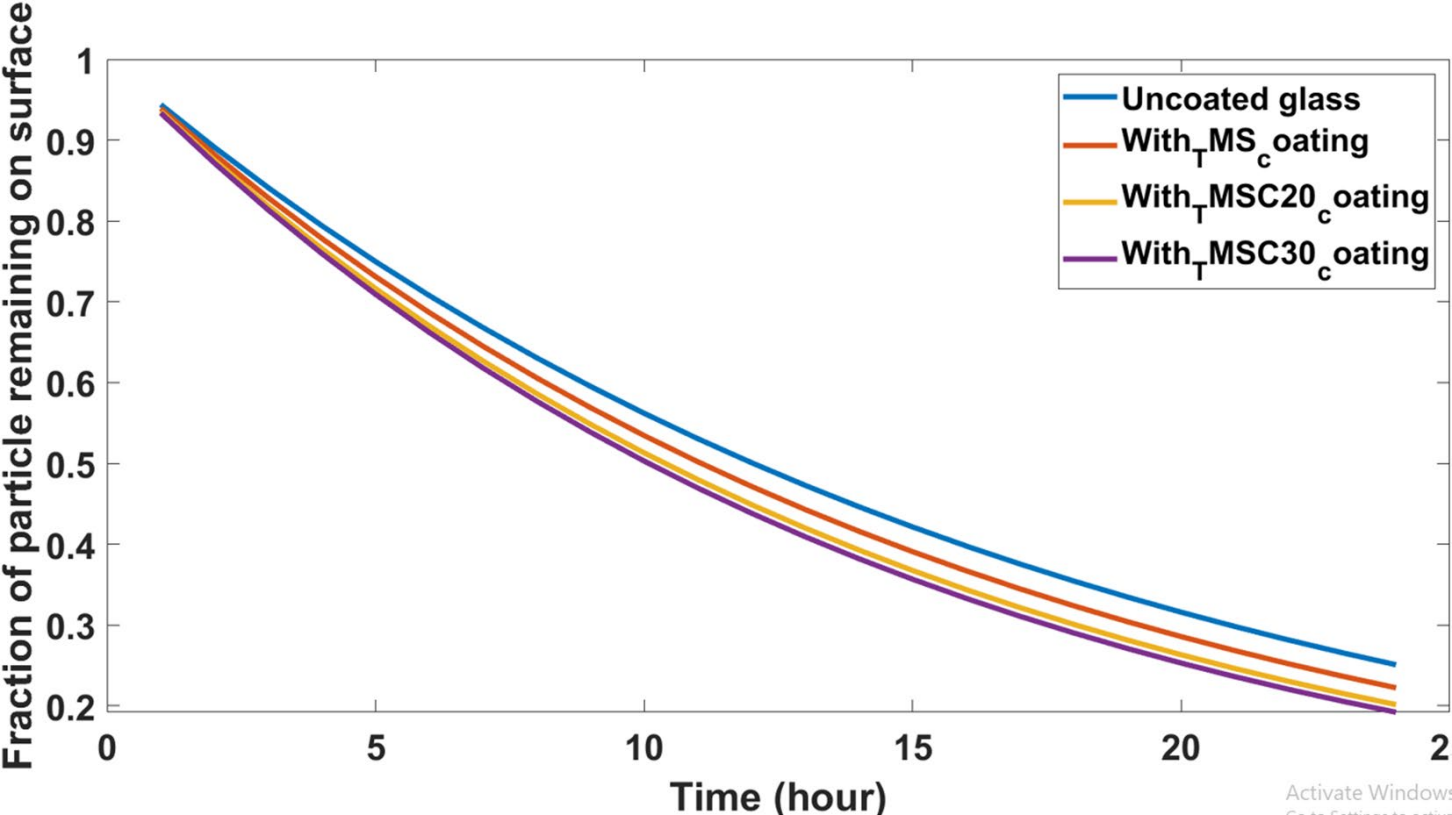
Fraction of particle remaining after rainfall over 24 h.

As the coatings have low surface free energy with high water contact angle, the removal rate of dust particle on coated surface is much higher than the bare glass as shown in Fig. 10.

**Fig. 11:**
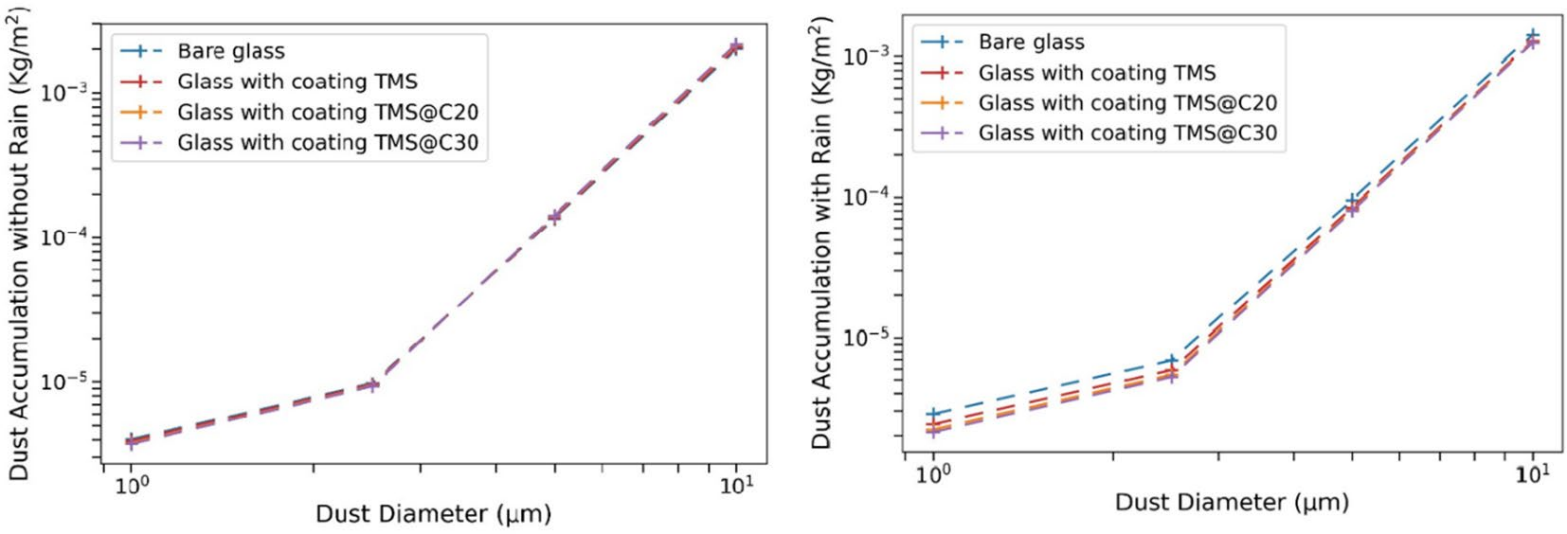
Dust accumulation with and without presence of rainfall.

**Fig. 12:**
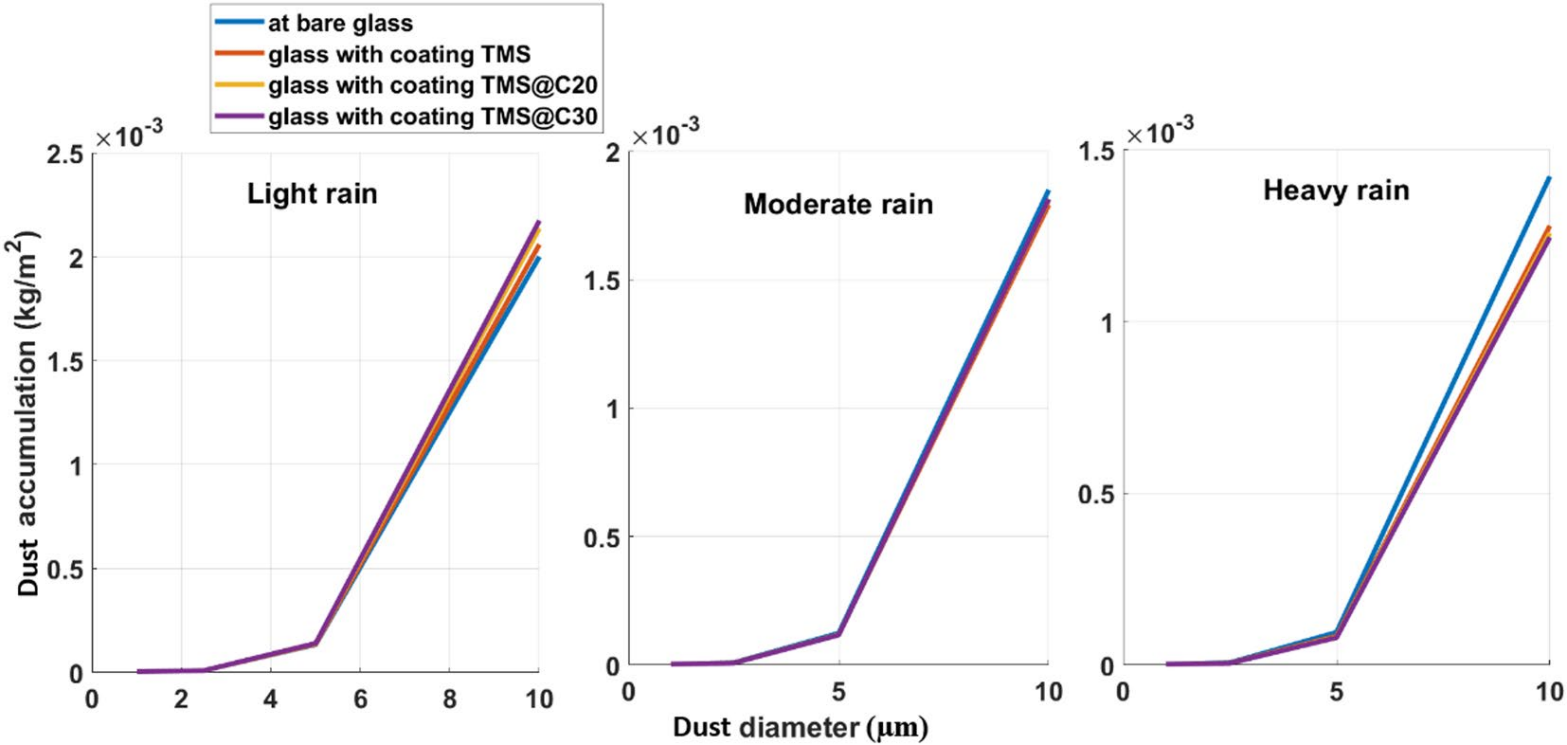
Dust accumulation for different intensity rainfall.

In absence of rainfall, accumulation of dust particle of smaller diameter (< 5 μm) on coated and uncoated glass is almost the same whereas for larger diameter particles (> 5 μm) accumulation is higher for TMS@C30 than uncoated glass (Fig. 11). As the removal rate of dust in presence of rainfall is much higher in case of coated glass, the accumulation of dust is lower on coated glass surface although the deposition rate is high (Fig. 11). It is observed from Fig. 12 that at low rain or drizzle, mass accumulation of dust is maximum on TMS@C30 coated sample, at moderate precipitation, phenomenon reverses, whereas for heavy rain results in complete wash-off of the dust particle from coated glass.

### Results from field experimentation

**Fig. 13:**
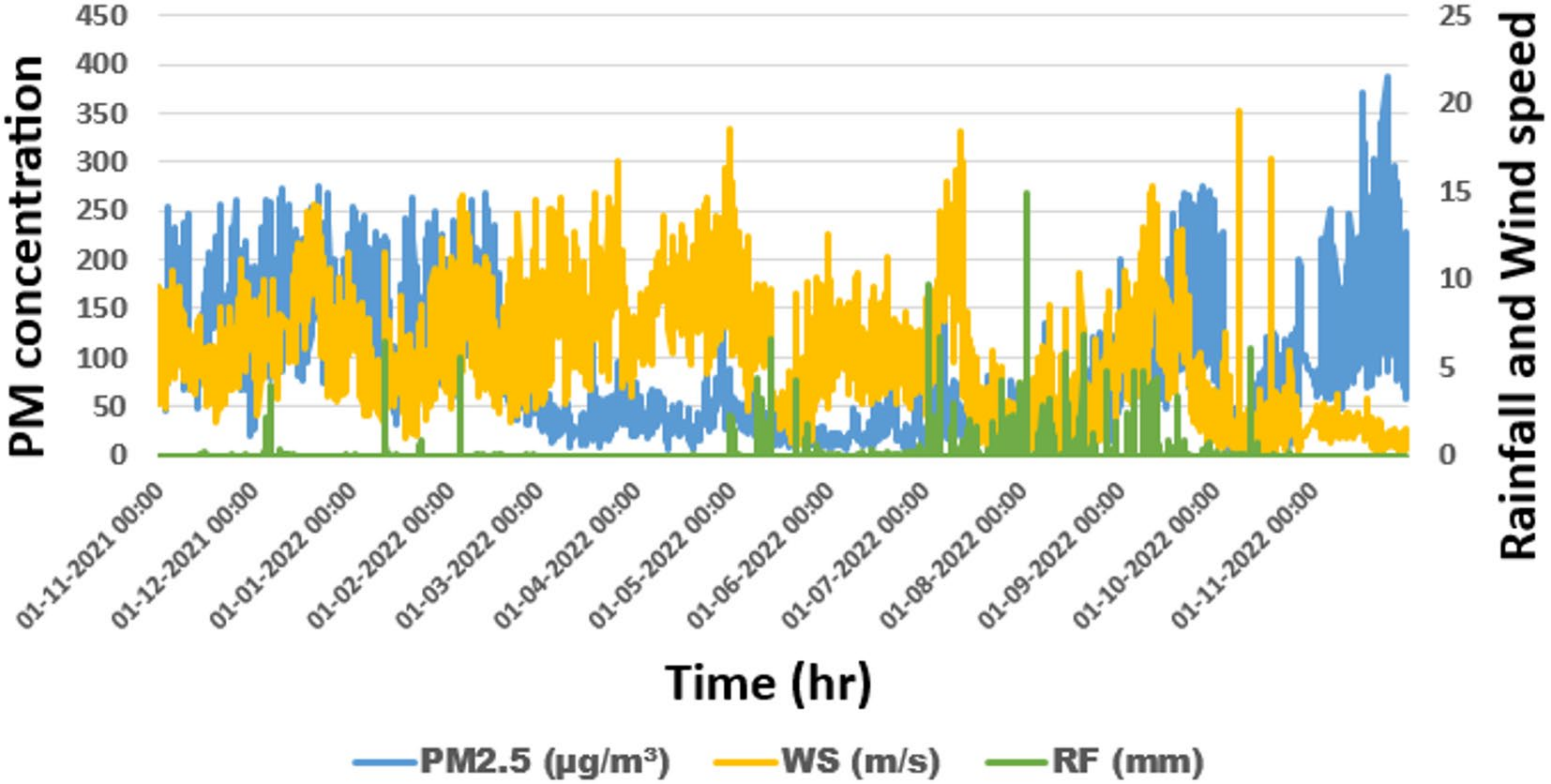
Daily weather data from November, 2021 to November, 2022.

**Fig. 14:**
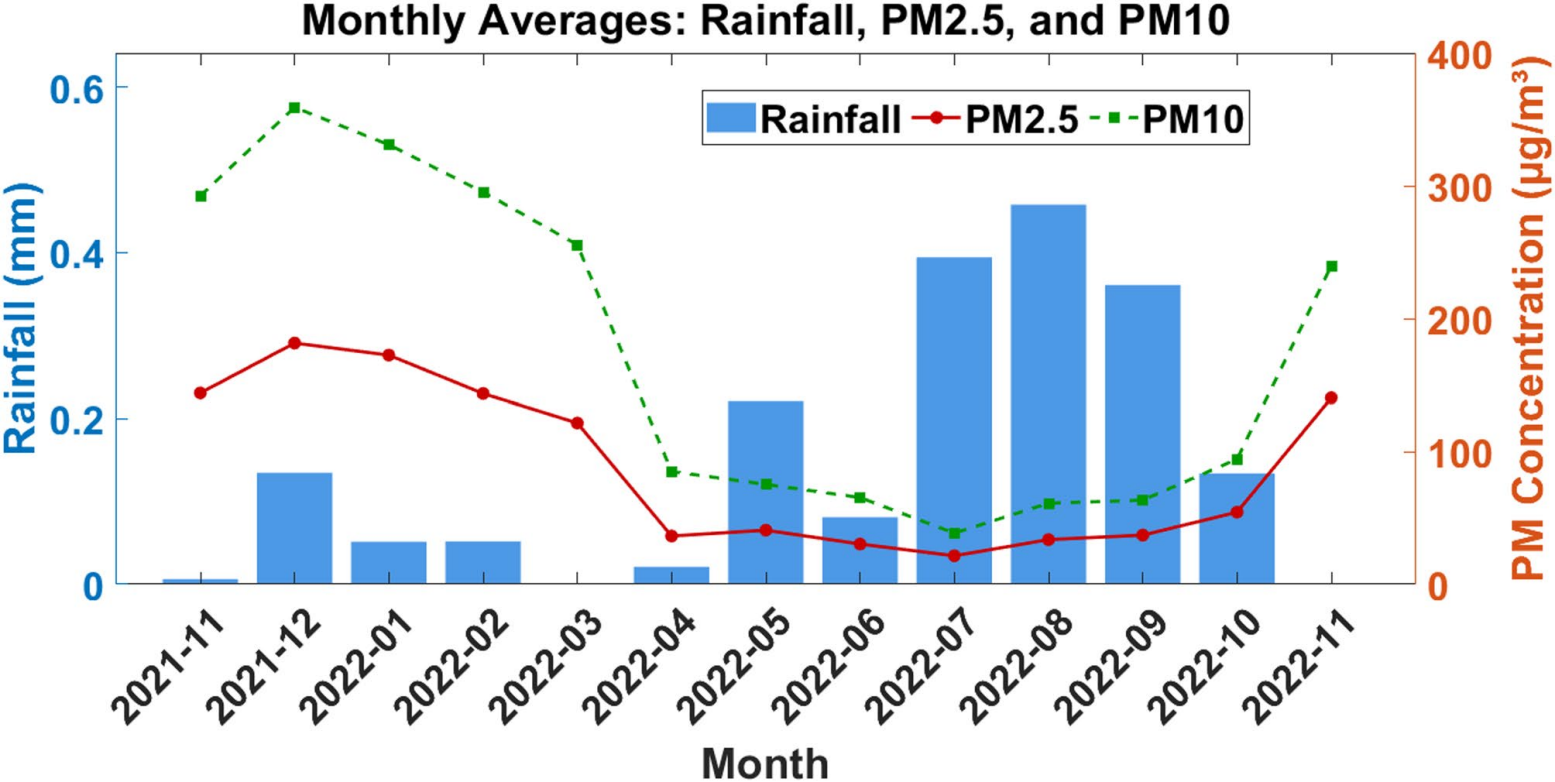
Daily average rainfall and PM concentration data.

**Fig. 15:**
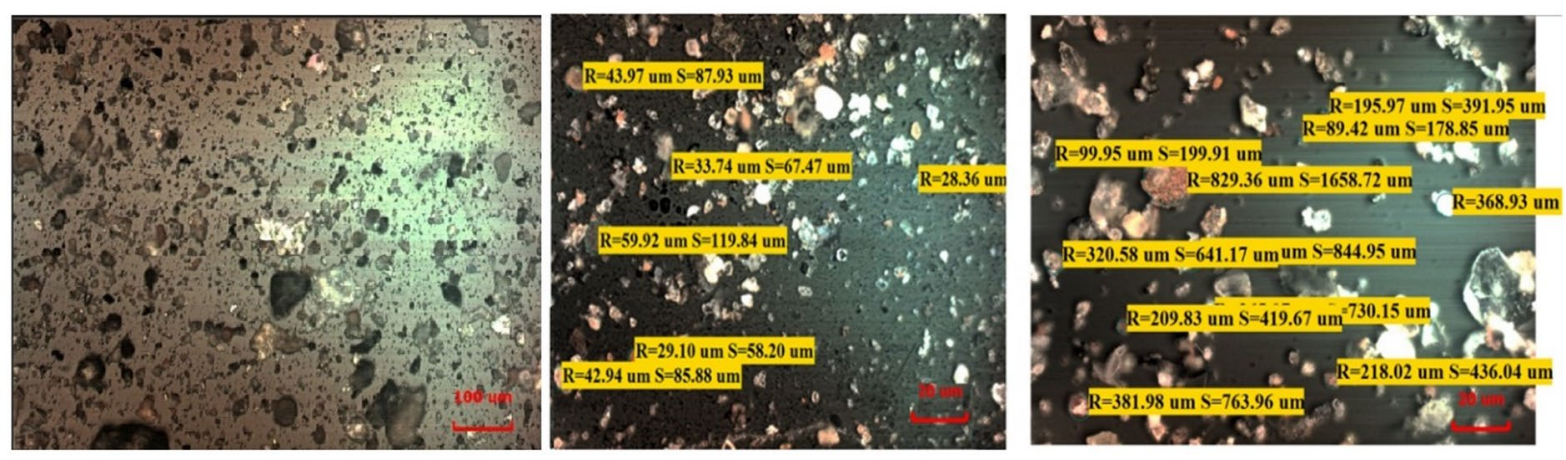
Seasonal variation of particle size in test location: summer *(left)*, spring *(middle)*, winter *(right)*.

The variation of weather parameters throughout the year is presented in Fig. 13. From Fig. 14, it is observed that, average monthly PM concentration during November, 2021 to March, 2022, is sufficiently high (> 300 µg/m^3^) whereas rainfall is quite low which can increase the PV soiling rate by wet deposition. In the subsequent period of the year, high rainfall scavenges the ambient particulate matters. Observed seasonal change of particle diameter has been depicted in Fig. 15. It is seen that in summer the mean of the particle size is 12 μm, in spring it is 35 μm and in winter it is 145 μm.

**Fig. 16:**
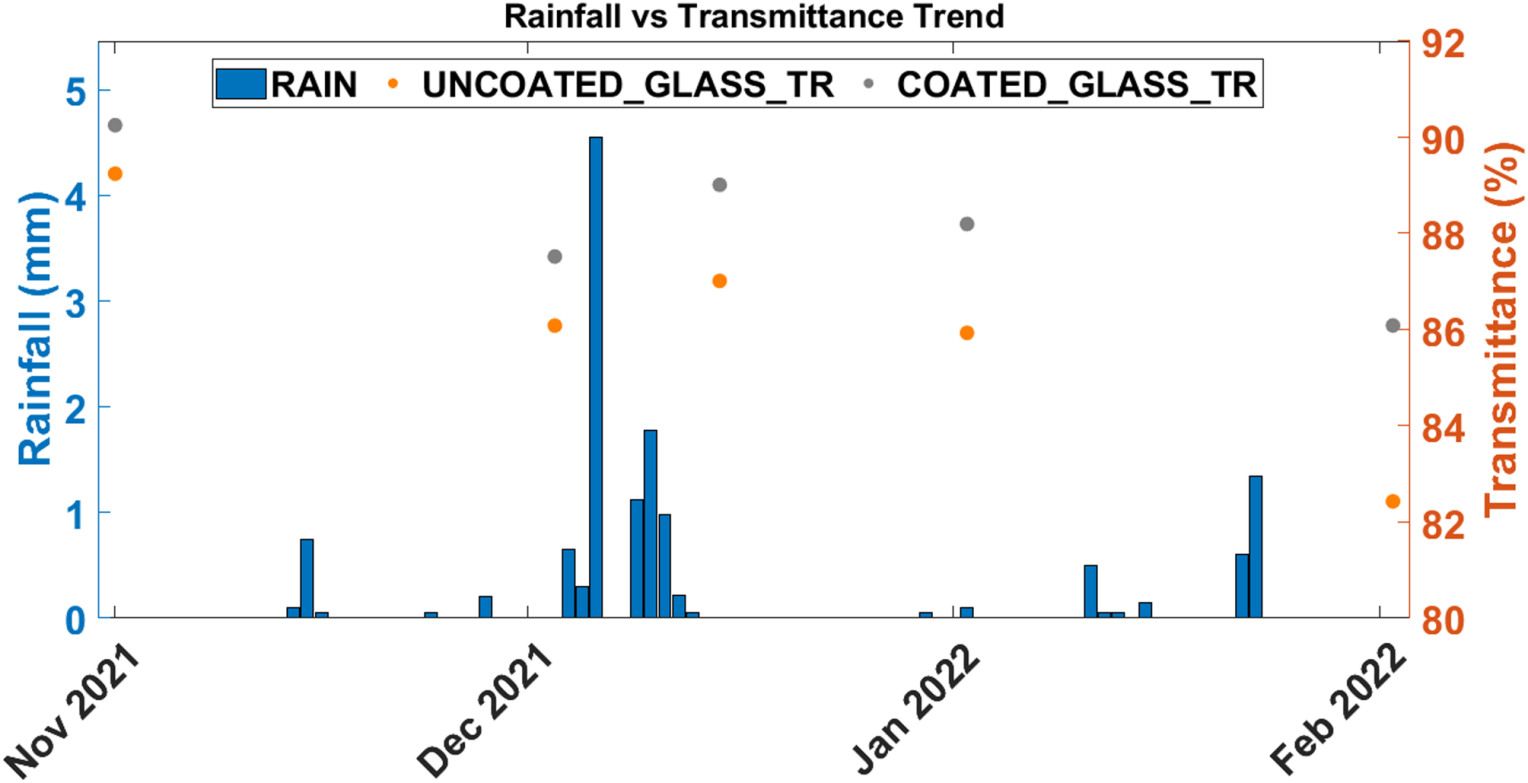
Effect of rainfall on transmittance variation of coated and uncoated glass.

**Fig. 17:**
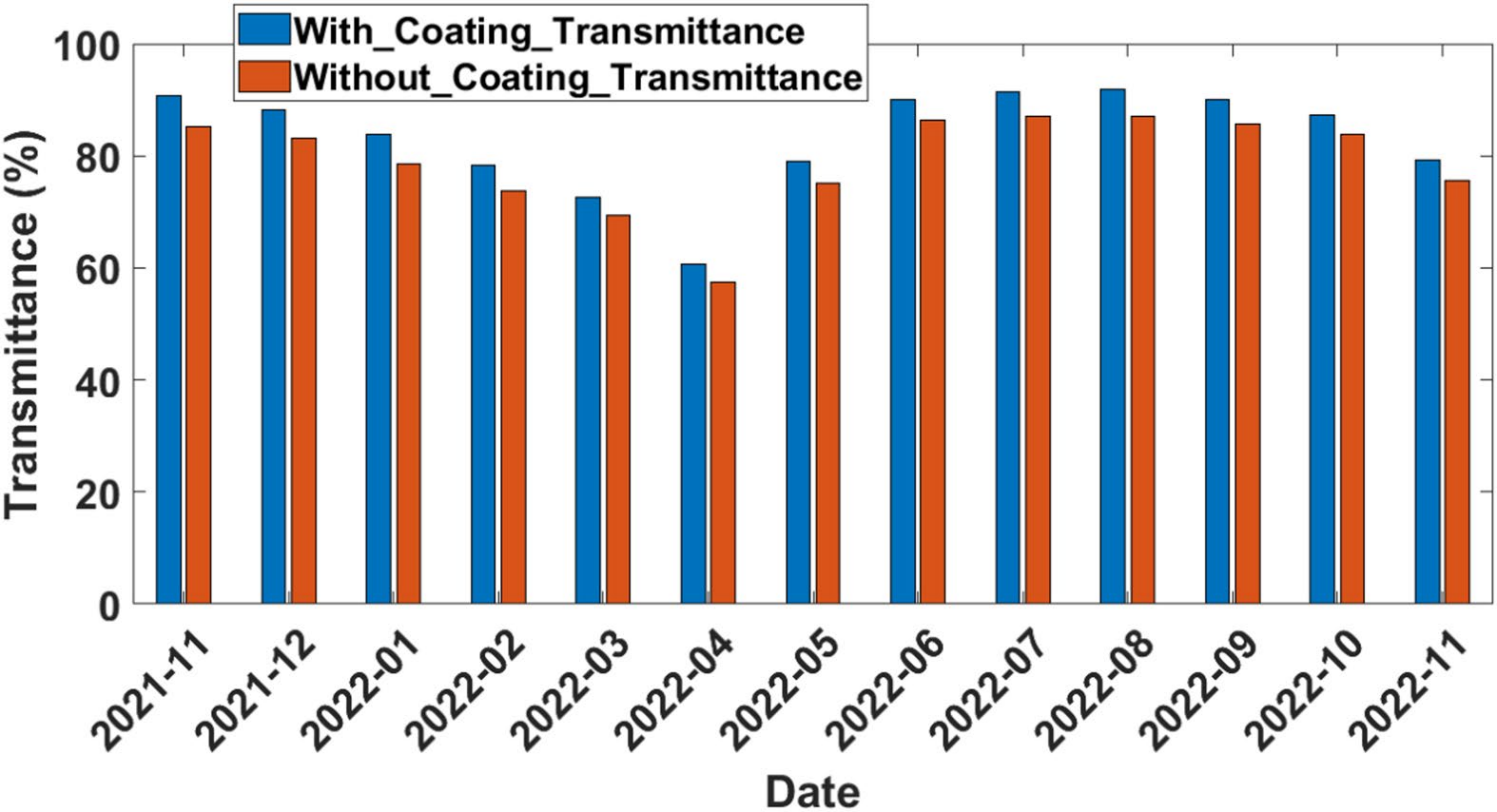
Monthly average of transmittance over one year.

In the month of February, transmittance of both coated and uncoated glass coupons are measured before and after rainfall. It is found that due to high PM concentration, low wind speed, dust accumulation is high on both the glass coupons (uncoated and coated) and hence the transmittance is found as 86% and 87% respectively on 3/12/2021. After the consecutive rainfall from 6/12/2021, as evidenced in Fig. 16, the same has increased upto 89% for coated glass whereas it is only 87% for bare glass. The average transmittance of coated and uncoated glass for every month over the year is presented in Fig. 17.

Glass samples with and without coating are kept for one year at outdoor condition and weighed periodically to measure the amount of dust deposition. Variation of cumulative dust accumulation for different seasons is presented in Table 5. The values are validated with the developed model which explores a good match.

**Table 5:** Amount of deposition variation on coated surface.

Time/season	Dust over uncoated glass (mg) (Measured)	Dust over coated glass (mg) (Measured)	Dust over uncoated glass (mg) (Calculated)	Dust over coated glass (mg) (Calculated)
18/11/2021 (Autumn)	2.5	0.3	2.43	0.40
10/1/2022 (winter)	5.3	0.7	4.98	0.62
25/3/2022 (spring)	4.7	1.3	4.56	0.98
7/6/2022 (Summer)	1.6	0.01	1.49	0.009
6/9/2022 (Monsoon)	0.2	0	0.21	0.00008

**Fig. 18:**
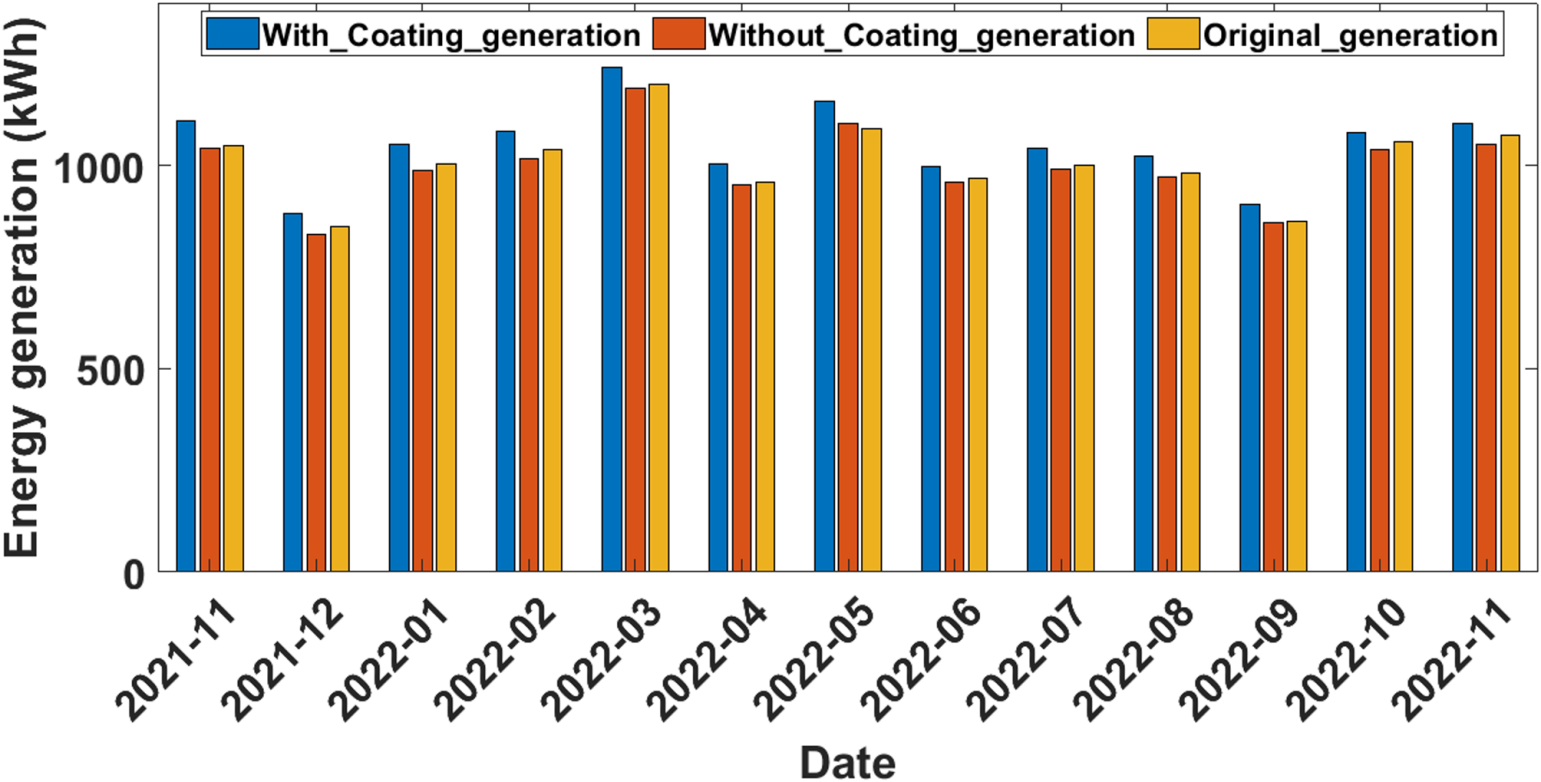
Generation estimation of a 10kWp PV plant with coated and uncoated glass.

**Fig. 19:**
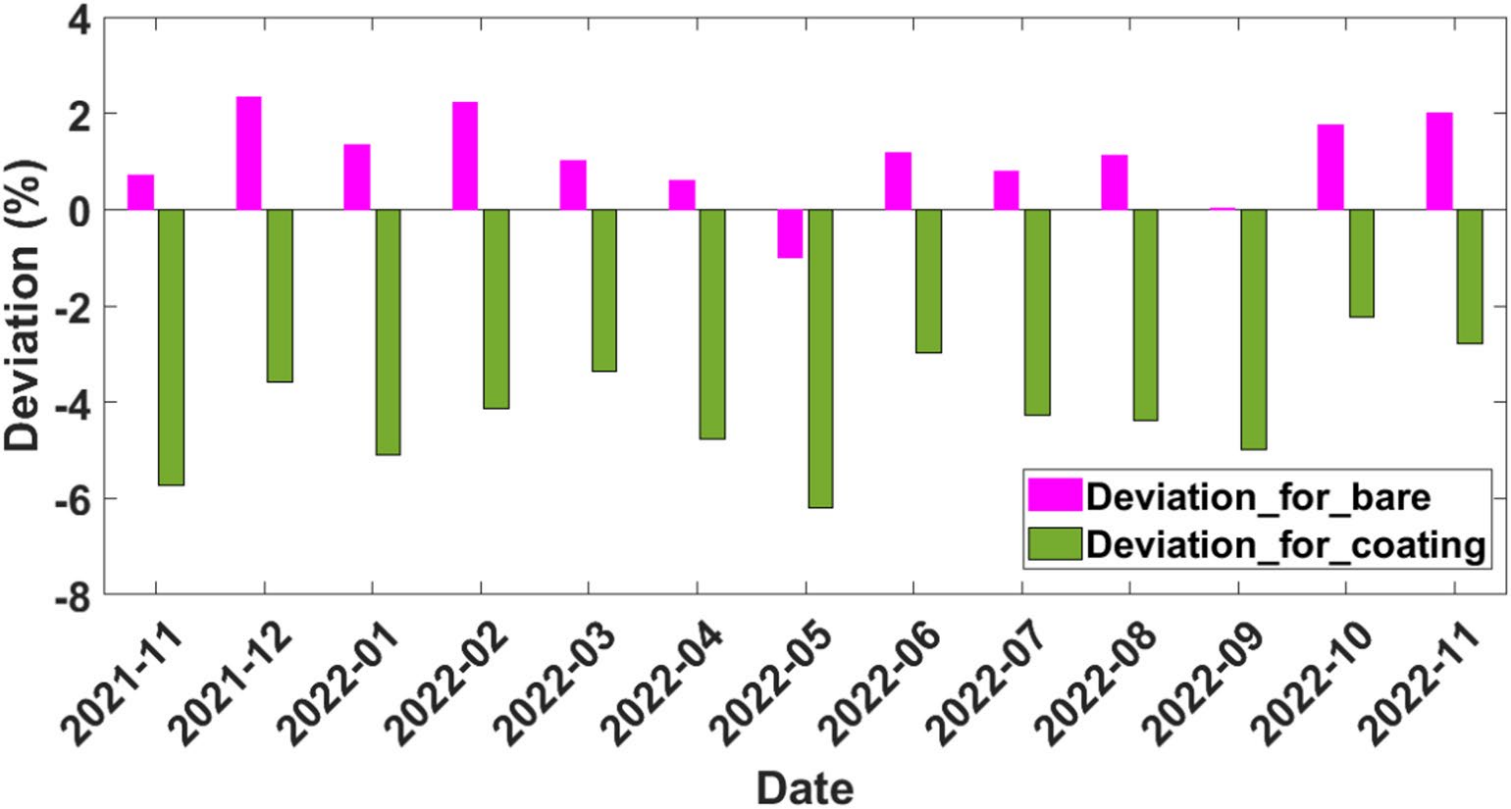
Estimated generation deviation.

Considering weather parameters (taken from Figs. 13 and 14) and transmittance variation of coated and bare glass under natural dust deposition and cleaning, a 10kWp solar PV power plant installed at IIEST, Shibpur (latitude: 22.5552° N) is used to estimate the energy yield for one year as mentioned. These estimated values are compared with the actual measured generation of plant with uncoated glass modules and presented in Fig. 18. It is observed that the estimated energy yield of a plant with developed coated glass module enhances by maximum of 6.2% with respect to measured value of the plant with uncoated glass (Fig. 19). This measured value is much closer (± 2%) to estimated result of plant without antisoiling coated module. From Fig. 16, it is observed that during December, 2021, frequent rainfall has occurred resulting in more cleaning of coated glass. This has increased the generation in coated glass plant January, 2022 as compared to uncoated glass plant (Fig. 18) and the deviation at that time seen to be of 5.1% (Fig. 19). Due to low and no rain during January-March, 2022, this deviation came down to 3.35% in March, 2022. During May, 2022 PM concentration is less and rainfall is high which establishes a high generation and high deviation. In the subsequent months low PM concentration, and high rainfall causes moderately high yield and hence deviation.

## Economic analysis

Cost benefit analysis is performed for the same 10kWp roof top PV plant in annual basis. The plant consists of 40 numbers of PV modules of 250Wp each capacity. Annual energy generation of the plant is 15 MWh (taking into account that 4kWh of generation is possible from 1kWp PV plant in a day), electricity tariff of Rs. 8.0 per kWh, soiling conditions are moderate at test location. Cleaning frequency is taken as 12 times/year. Capital cost for coating with newly developed material is Rs. 8000/-. Cost of cleaning water, its pumping and labour cost is Rs. 6736/-, total coming out to be Rs. 14736/-. The coated module shows ~ 6% generation increment, which reveals that there is an annual excess generation of 900kWh, which costs Rs. 7200/-. Hence, the return of investment (ROI) period is ~ 2 years. Rather than coating, for this particular plant, the use of robotic cleaning system will result in CAPEX of ~ Rs. 20,000/- as well as it requires more water and maintenance cost of robot, which will ultimately increase ROI. Manual cleaning will also require more water with sprinkler constructional cost and labor cost, which will ultimately increase ROI to 3–4 years.

## Conclusion

In this paper, three types of polysiloxane-based antisoiling coatings are developed. Out of those, TMS@C30 is seen to have the best performance since it has refractive index 1.37, water contact angle 97°, and pencil hardness 6 H. Standard accelerated UV test results in degradation in the order of 1.2% for TMS@C30. A physics-based model is used to simulate the dust accumulation and effect of water-based cleaning on various developed coated glasses alongwith non-coated glass. Simulation results demonstrate that in absence of rainfall, accumulation of smaller particles (< 5 μm) is closer for coated and uncoated glass, but for bigger particles (≥ 5 μm), coated glass holds more numbers. However, in presence of rainforce the overall dust removal is much higher in coated glass than bare one which establishes the impact of water on the developed coating.

The above results are validated by one-year long field experimentation with natural deposition and cleaning which has been carried out at IIEST, Shibpur with TMS@C30 coated glass and with uncoated one. Observation shows that although after one month of commencement of experimentation, transmittance of coated glass is 1.64% higher than uncoated one, after rainfall the same exceeds to 2.24%. This has been extended to energy generation estimation of a 10kWp PV power plant where it has also been established that a plant with coated glass can generate ~ 6% more energy in presence of rain/water-based cleaning. With this enhanced generation, a cost benefit analysis has also been done which shows that the ROI period is about 2 years whereas for robotic cleaning or manual cleaning, it increases to 3–4 years with increased usage of water.

## Data Availability

Data will be made avaiable upon request to corresponding author.
